# Secondary hyperparathyroidism among Nigerians with chronic kidney disease

**DOI:** 10.4314/ahs.v18i2.30

**Published:** 2018-06

**Authors:** Zumnan M Gimba, Esala E Abene, Oche O O Agbaji, Emmanuel I Agaba

**Affiliations:** 1 Nephrology Division, Department of Medicine, Jos University Teaching Hospital, Nigeria; 2 Department of Medicine, University of Jos

**Keywords:** Secondary hyperparathyroidism, chronic kidney disease, intact parathyroid hormone, hypocalcaemia, hyperphosphataemia, elevated alkaline phosphatase

## Abstract

**Backround:**

Secondary hyperparathyroidism (SHPT) is a manifestation of chronic kidney disease mineral bone disorder (CKD-MBD). SHPT is common in patients with chronic kidney disease (CKD) and is associated with significant morbidity and mortality.

**Methods:**

A cross- sectional descriptive study involving 230 patients with CKD.

**Results:**

The mean age of the study population was 44.17±15.24 years. The median intact parathyroid hormone and alkaline phosphatase levels were 96pg/ml (range 4–953pg/ml) and 88 iu/l (range 10–800 iu/l) respectively. The mean (with standard deviation) calcium, serum phosphate, calcium phosphate product and haemoglobin levels were 2.22±0.29mmol/l, 1.8±0.62mmol/l, 3.94±1.42mmol^2^/l^2^ and 9.90±1.87g/dl respectively. Majority of patients had advanced CKD with 70.3% of patients in stage G5. The prevalence rates of SHPT, hypocalcaemia, hyperphosphataemia, elevated alkaline phosphatase and elevated calcium phosphate product were 55.2%, 34.8%, 66.1%, 42.2% and 25.2% respectively.

Univariate analysis revealed that SHPT was associated with hypocalcaemia, hyperphosphataemia, elevated alkaline phosphatase, proteinuria, anaemia, hypertension, left ventricular hypertrophy and stage of kidney disease; being worse with advancing kidney disease. Independently associated with SHPT were hypocalcaemia (OR=4.84), hyperphosphataemia (OR=3.06), and elevated alkaline phosphatase (OR=2.04).

**Conclusion:**

The prevalence of SHPT in CKD is high, occurs early and is independently associated with hypocalcaemia, hyperphosphataemia and elevated alkaline phosphatase. The prevalence of SHPT also increases with worsening renal function.

## Introduction

Chronic kidney disease (CKD) affects between 5 to 10% of the population worldwide and is associated with significant morbidity and mortality mainly from cardiovascular causes^1^–^3^. A number of endocrine and metabolic disorders complicate CKD, one of which is secondary hyperparathyroidism (SHPT). SHPT, a component of Chronic Kidney Disease-Mineral and Bone Disorder (CKD-MBD) is defined by the presence of elevated parathyroid hormone (PTH) level as well as abnormalities in mineral and bone metabolism^1^,^4^–^6^. There is increasing prevalence of SHPT across declining glomerular filtration rates (GFR), and this has been identified as a risk factor for morbidity and mortality as it promotes vascular calcification among others^7^–^9^. The effects of SHPT are manifested in different parts of the body, causing bone pains, athralgia, muscle weakness, pruritus, bony deformities and increased fracture risk^4^. The bone marrow fibrosis that can occur contributes to anaemia and erythropoietin resistance^9^. Thus, elevated PTH is considered a uremic toxin^10^,^11^.

SHPT occurs in 28.9% of CKD patients in the Philippines, 45% in Iranian patients and 55% of American patients^12^–^14^. There are only few studies concerning this condition in Africa^15^–^17^. SHPT occurs in 17–28. 1% of Libyan CKD patients^16^,^17^. Previous studies in Nigeria utilising a small sample found SHPT in 18.1% of patients in South West Nigeria^15^. Another study in South Eastern Nigeria using only radiological features of renal osteodystrophy found a low prevalence of 3.3%^18^. There is therefore the need for a larger study evaluating the burden of SHPT in patients with CKD.

In this study, we hypothesise that SHPT is rare among Nigerians with CKD. We describe the prevalence of SHPT and its clinical correlates in a cross-sectional study involving CKD patients presenting in a teaching hospital in North Central Nigeria.

## Patients and methods

### Study design

This was a hospital based cross- sectional descriptive study, conducted between August 2011 and September 2012. It was carried out in the Nephrology and Hypertension Clinic and medical wards of the Jos University Teaching Hospital (JUTH).

### Study population

Patients 18 years and above attending the Nephrology and Hypertension Clinic or who were admitted into the medical wards of JUTH and confirmed to have CKD were recruited using convenience sampling technique. CKD patients already on treatment for SHPT as well as pregnant CKD patients were excluded from the study.

### Data collection

Demographic and clinical data were entered into a case report form. Information obtained included age, sex, weight, height, aetiology of CKD, history of body weakness, pruritus, and bone tenderness. Height and Weight were measured in metres and kilograms using a stadiometer and weighing scale with patients in light clothing, without head gears and without shoes. Body mass index (BMI) was calculated using the Quetelet index^19^. Blood pressures were measured according to standard procedures^20^.

Blood samples were obtained from the forearm vein of each subject for intact parathyroid hormone (iPTH), haemoglobin, serum creatinine, albumin, calcium, phosphate, alkaline phosphatase. In addition, a 12 lead electrocardiograph tracing, abdominal ultrasonography for renal sizes, as well as spot urine protein creatinine ratio determination was performed on all patients. Estimated glomerular filtration rate (eGFR) was calculated for each patient using the chronic kidney disease-epidemiology collaboration (CKD-EPI) formula^21^.

### Laboratory methods

Enzyme linked immunosorbent assay (ELISA) kits (Diagnostic Automation Inc, California, USA) were used for the determination of biologically active intact 84 amino acid chain of PTH. In this reaction, iPTH binds to a polyclonal antibody that has been labelled with an indicator and binds only to the biologically active 84 amino chain of PTH. The colour change by the indicator is proportional to the concentration of iPTH with absorbance read at 405nM. Concentrations of iPTH are obtained using a dose response curve of absorbance unit versus concentration.

Biochemical analysis for the determination of serum calcium, phosphate, alkaline phosphatase, albumin, and serum lipids were all determined using an autoanalyser (Roche Diagnostics GmbH, Mannheim Germany).

In this study secondary hyperparathyroidism was defined as iPTH≥65pg/ml, hyperphosphataemia was defined as serum phosphate above 1.4mmol/l, hypocalcaemia was defined as corrected serum calcium level below 2.1mmol/l, elevated calcium and phosphate product (CaXP) was defined as values greater than 4.5mmol^2^/l^2^ while elevated alkaline phosphatase was serum total alkaline phosphatase above 96 iu/ml. Proteinuria was defined as the presence trace amounts of proteins in urine and above on conventional urinalysis strip (equivalent to 15mg/dl and above). Left ventricular hypertrophy was defined based on the Sokolow-Lyon criteria^22^.

### Ethical consideration

The human research and ethics committee of the Jos University Teaching Hospital (JUTH) approved the study. All patients gave informed consent before recruitment. Data was anonymised by removing all patient identifiers. Data was kept safe until analysis.

### Statistical analysis

Epi - Info version 3.5.4 (CDC Atlanta USA) software was used for data analysis. Continuous variables with normal distribution were expressed as means ± SD while variables not normally distributed were expressed as median with ranges. Categorical variables were expressed as frequencies and percentages. Chi-square test was used to determine significance of association between categorical variables. Fisher exact test was used when there were less than 5 observations. Student's t- test was used to compare means. Multivariate analysis was used to identify variables independently associated with hyperparathyroidism. In this case, variables with a p value of <0.25 on univariate analysis were entered into a multiple logistic regression model to determine their independent association with secondary hyperparathyroidism with backward elimination done to adjust for confounders. The use of p <0.25 is based on documented findings that the use of p<0.05 often fails to identify all variables known to be important^23^. Level of significance at p < 0.05 was used throughout.

## Results

**Figure 1:**
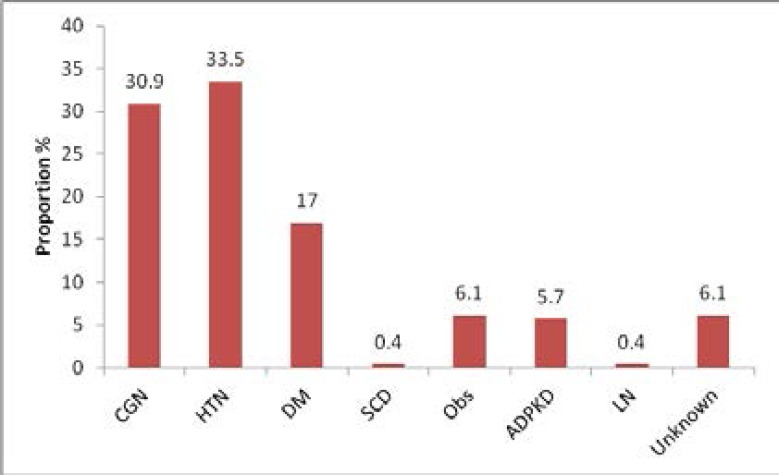
Bar graph showing aetiology of CKD in the study population

A total of 230 CKD patients were enrolled for the study, one hundred and twenty six of these patients (54.8%) were males while one hundred and four patients (45.2%) were females with a male to female ratio of 1.2:1. The mean age of the study population was 44± 15 years. All patients were Black. Majority of these patients (33.5%) had hypertension as the primary cause of renal disease, followed by 30.9% with chronic glomerulonephritis and 17% with diabetes mellitus (Figure 1).

**Figure 2:**
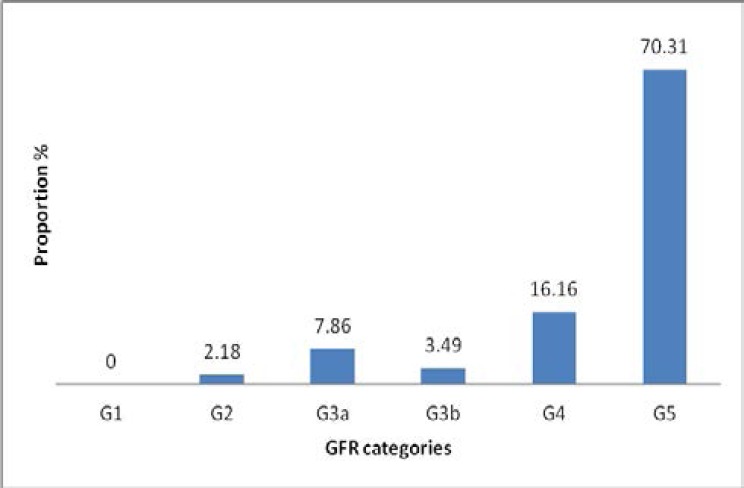
Bar graph showing CKD stages based on GFR categories

In addition, majority of the patients had advanced renal disease with 70.3% of them in CKD G5 and 16.1% in CKD G4 (Figure 2). The socio-demographic and clinical characteristics of the patients are as shown in Tables 1 and 2 respectively.

**Table 1:** Socio demographic data of the study population

Variable	Total (n=230)	Females (n=104)	Males (n=126)	p value
**Age, years**	44.17 ±15.60	41.04±15.24	46.73±15.45	0.005
**Weight (kg)**	65.8±12.8	63.7±14.0	67.5±11.5	0.003
**Height (M)**	1.63±0.07	1.59±0.05	1.67±0.06	<0.0001
**BMI** (Kg/M^2^)	24.44±4.23	24.93 ±4.86	24.02±3.58	0.27
**Education (n, %)**				0.01
Uneducated	33 (14.4)	22(21.2)	11 (8.7)	
Primary	51 (22.2)	27 (26.0)	24 (19.0)	
Secondary	66 (28.7)	27 (26.0)	39 (31.0)	
Tertiary and above	80 (34.8)	28 (27.0)	42 (41.2)	
**Marital status (n, %)**				<0.0001
Married	152 (66.1)	56 (53.8)	96 (76.2)	
Single	51 (22.2)	25 (24.0)	26 (20.6)	
Divorced	6 (2.6)	5 (4.8)	1 (0.8)	
Widowed	21 (9.1)	18 (17.3)	3 (2.4)	
**Occupation (n, %)**				<0.0001
Employed	122 (53.0)	38 (36.5)	84 (66.7)	
Unemployed	79(34.4)	48 (46.15)	31 (24.60)	
Student	29 (12.6)	18 (17.3)	11 (8.7)	
**Residence (n, %)**				
Rural	56 (24.3)	26 (25.0)	30 (23.8)	0.41
Urban	174 (75.7)	78 (75.0)	96 (76.2)	

Employed = civil servant, private employment, self employment), BMI= Body Mass Index. Values are in means±Standard Deviation or numbers (%), p values <0.05 are significant

**Table 2:** Biochemical parameters of the study population

Variable	Total (n=230)	Females (n=104)	Males (n=126)	P value
Proteinuria (n,%)	186 (80.9)	86 (82.7)	100 (79.4)	0.52
UPCR (mg/mg) median with range	0.8 0.02–39.0	0.9 0.03–5.90	0.70.02–39.00	0.95
Creatinine (µmol/l) median with IQR	564.50316.0–1000.0	623.50324.0–1000.0	539.0 308.0–981.0	0.45
eGFR((ml/min/1.73m^2^) median with IQR	9.45.5–18.0	7.6 4.7–15.2	10.9 6.5–21.7	0.0003
Haemoglobin (g/dl)	9.90±1.87	9.82±1.87	9.97±1.87	0.54
Serum albumin (g/dl)	35.95±7.76	35.45±6.83	36.36±8.45	0.77
Calcium corr. (mmol/l)	2.22±0.29	2.21±0.25	2.24±0.33	0.51
Serum phosphate (mmol/l)	1.8±0.62	1.81±0.83	1.83±0.83	0.64
CaXP (mmol^2^/l^2^)	3.94±1.42	3.97±1.33	3.91±1.49	0.75
Serum ALP (iu/l)	8810-800	8510-221	9025-800	0.27
Serum iPTH (pg/ml)	964-953	964-953	944-953	0.35
Total cholesterol (mmol/l)	4.81±1.61	5.06±1.49	4.60±1.82	0.004
HDL (mmol/l)	1.06±0.50	1.08±0.58	1.04±0.42	0.71
LDL (mmol/l)	2.81±1.34	2.98±1.27	2.67±1.38	0.08
ECG (n,%)				0.51*
Normal	112 (49.3)	46 (45.1)	66 (52.8)	
LVH	109 (52.0)	53 (52.0)	56 (44.8)	
Low voltages	6 (2.6)	3 (2.9)	3 (2.4)	
Kidney sizes (cm)				
Right	9.67±2.10	9.40±2.02	9.90±2.15	0.07
Left	9.76±2.32	9.35±2.09	10.09±2.46	0.01

UPCR- Urine Protein Creatinine Ratio, eGFR- estimated Glomerular Filtration Rate, Calcium corr.-corrected calcium, CaXP- Calcium Phosphate product, ALP- Alkaline phosphatase, iPTH-intact parathyroid hormone, HDL-high density lipoprotein, LDL- low density lipoprotein, ECG-

Table 3 shows the prevalence of SHPT, hypocalcaemia, hyperphosphataemia, elevated alkaline phosphatase, elevated calcium phosphate product as well as anaemia in the study population. All of these were compared between male and female patients. The prevalence of SHPT was 55.2% overall with no significant difference between males and females (p = 0.70).

**Table 3:** Prevalence of shpt and other markers of CKD-MBD

Variable	Total (n=230)	Females (n=104)	Males (n= 126)	p value
SHPT (n,%)	127(55.2)	56(53.8)	71 (56.3)	0.70
Hypocalcaemia (n,%)	80 (34.8)	36(34.6)	44 (34.9)	0.96
Hyperphosphataemia (n,%)	152 (66.1)	74 (71.2)	78 (61.9)	0.14
Elevated ALP (n,%)	97 (42.2)	39 (37.5)	58 (46.0)	0.19
Elevated CaXP (n,%)	58 (25.2)	29 (27.9)	29 (23.0)	0.39
Anaemia (n,%)	187 (81.3)	84 (80.8)	103 (81.7)	0.85

SHPT- secondary hyperparathyroidism, ALP- alkaline phosphatase, CaXP- calcium phosphate product

**Figure 3:**
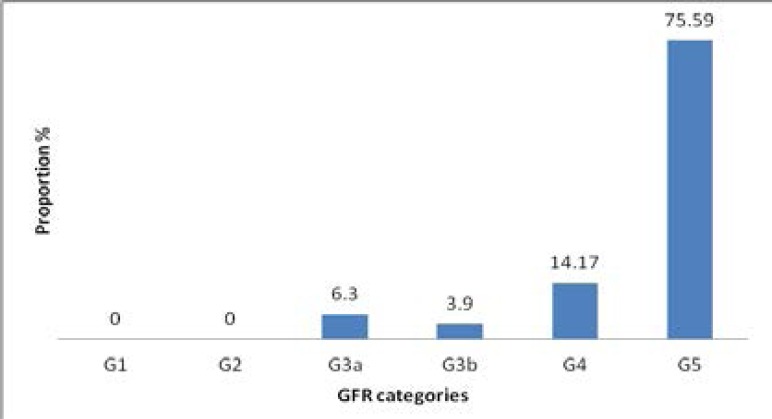
Bar graph showing prevalence of SHPT across different CKD stages

**Figure 4:**
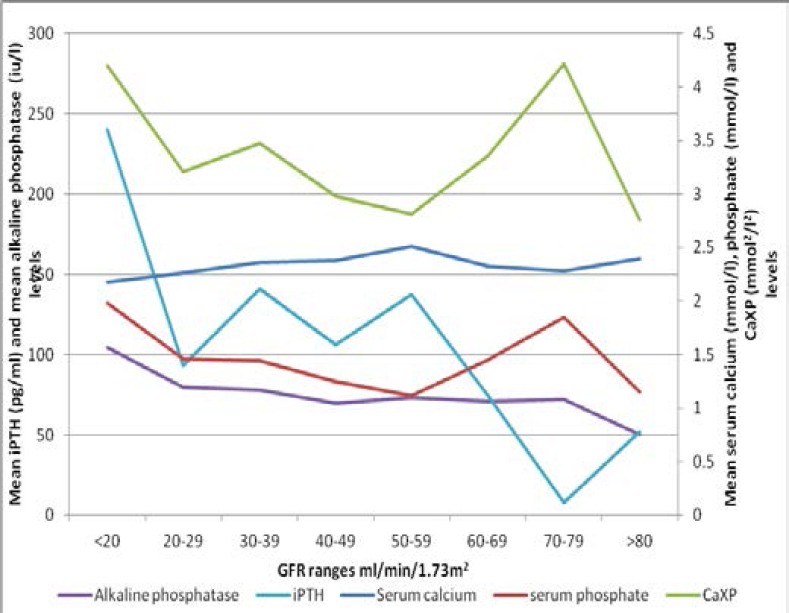
Mean values of calcium, phosphate, CaXP, iPTH and alkaline phosphatase levels across GFR ranges

Figure 3 is a bar graph showing the prevalence of SHPT across CKD stages, with SHPT occurring as early as in CKD G3a and is highest in CKD G5. Additionally, with worsening of the eGFR, the higher the mean values of phosphate, alkaline phosphatase, PTH and CaXP while lower mean values of calcium were observed with worsening of eGFR (Figure 4).

The various factors associated with SHPT on univariate analysis are as shown in Table 4. SHPT was found to be associated with hypertension, proteinuria, low estimated glomerular filtration rate, left ventricular hypertrophy, hypocalcaemia, hyperphosphataemia, elevated alkaline phosphatase, elevated calcium phosphate product and anaemia. SHPT was not found to be associated with its clinical features as well as the aetiology of the CKD. However on multivariate analysis, only hyperphosphataemia, hypocalcaemia and elevated alkaline phosphatase levels were found to be independently associated with SHPT and are shown in Table 5.

**Table 4:** univariate analysis of factors associated with SHPT

VARIABLE	SHPT	NO SHPT	p value
Age (mean ± SD)	44.4± 15.7	43.8± 15.4	0.77
Sex	127	103	0.35
Females (n, %)	56 (53.8)	48 (46.2)	
Males (n, %)	71 (56.3)	55 (43.7)	
Body weakness (n, %)	96 (75.6)	71 (68.9)	0.13
Body pains (n, %)	44 (34.6)	31 (30.1)	0.23
Pruritus (n, %)	37 (29.1)	27 (26.2)	0.31
Bone tenderness (n, %)	20 (15.7)	16 (15.5)	0.48
Scratch marks (n, %)	10 (7.9)	14 (13.6)	0.08
Recent fracture (n, %)	1 (0.8)	1 (1.0)	0.69*
Hypertension (n, %)	68 (53.5)	40 (38.8)	0.01
Proteinuria (n, %)	112 (88.2)	74 (71.8)	<0.001
eGFR mean(range)	14.7 (2.6–63.9)	20.0 (2.3–80.4)	0.001
Elevated Cholesterol (n, %)	17 (13.4)	14 (13.6)	0.48
Low HDL (n, %)	73 (57.5)	59 (57.3)	0.48
Elevated LDL (n, %)	15 (11.8)	14 (13.6)	0.34
Elevated Triglycerides (n, %)	35 (27.6)	21 (20.4)	0.10
LVH on ECG (n, %)	68 (54.8)	41 (39.8)	0.02
Hypocalcaemia (n, %)	67 (52.8)	13	<0.001
Hyperphosphataemia (n, %)	103 (80.3)	(12.6)	<0.001
Elevated ALP (n, %)	66 (52.0)	50 (48.5)	<0.001
Elevated CaXP (n, %)	37 (29.1)	31 (30.1)	0.06
Anaemia (n, %)	114 (89.8)	21 (20.4)	<0.001
Diabetic nephropathy (n, %)	23 (18.1)	16 (15.5)	0.30
Hypertension (n, %)	47 (37.0)	30 (29.1)	0.10
Chronic glomerulonephritis (n, %)	40 (31.5)	31 (30.1)	0.41

**Table 5:** factors independently associated with SHPT

Variable (Yes/No)	Odds ratio	95% Confidence interval	p value
Hypocalcaemia	5.41	2.66–10.99	<0.0001
Hyperphosphataemia	2.87	1.52–5.41	0.001
Elevated ALP	2.04	1.13–3.86	0.01

## Discussion

The prevalence of SHPT among patients with CKD in Jos was found to be high and occurred in 55.2% of subjects studied. This study also found high prevalence rates of hyperphosphataemia, hypocalcaemia, elevated alkaline phosphatase and anaemia. This is similar to that of Bhan et al^14^ who reported a prevalence of 55% among CKD patients in the United States of America. The prevalence of SHPT found in this study is higher than earlier smaller studies by Sanusi et al^15^ and Odenigbo and colleagues^18^, thus showing that SHPT is common among Nigerians CKD patients than previously thought. The role of the Black race in the development of SHPT could also explain this high prevalence. Studies by Gupta et al^24^ and Kalantar- Zadeh et al^25^ revealed that Black CKD patients have a 4.4 fold chance of developing SHPT than White CKD patients. It is therefore not surprising that a large proportion of the patients in this study have SHPT.

SHPT was also found to occur early with decreasing GFR levels and CKD categories. These findings are similar to those reported by Levin and colleagues^7^ in the SEEK study where SHPT occurred in up to 12% of CKD patients with GFR above 80ml/min/1.73m^2^. This is perhaps drawing attention to the fact that SHPT could possibly develop much earlier than GFR less than 60 ml/min/1.73m^2^ and thus should be sought for in CKD patients at an early stage.

This study found no association between parathyroid hormone levels on the one hand and clinical features of SHPT on the other. This could be because symptoms and signs of SHPT although common are non specific. In this study, the most common symptoms reported by the patients were body weakness, pruritus and bone pains with the least common feature being history of fractures; a similar picture was described by Seck et al^26^ in Senegal who found that pruritus and bone pains were the most common symptoms and fractures the least. This however differed from the findings by Sanusi et al^15^ where majority of their patients with CKD-MBD were asymptomatic. Clinical and biologic manifestations of secondary hyperparathyroidism are non-specific and accurate diagnosis is difficult in the absence of more specific diagnostic tests as is often the case in most resource poor settings like Nigeria.

The prevalence of hypocalcaemia, hyperphosphataemia, elevated alkaline phosphatase and elevated calcium phosphate product were all high in this study. These were all significantly associated with secondary hyperparathyroidism on univariate analysis. All of them, with the exception of elevated calcium phosphate product were independently associated with secondary hyperparathyroidism. This concurs with the finding of Sanusi et al^15^ where high prevalence of hypocalcaemia, hyperphosphataemia as well as increased alkaline phosphatase were seen in their patients with renal bone disease, which in a number of studies has been linked to increased mortality in patients with secondary hyperparathyroidism. Hypocalcaemia, hyperphosphataemia, elevated alkaline phosphatase and elevated calcium and phosphate product (CaXP) were also found to have higher prevalence rates with decreasing glomerular filtration rates. Thus the more severe the kidney damage, the higher the prevalence of these markers, a finding similar to that of Levin et al.^7^

To our knowledge, this the largest study on SHPT in Nigeria, which has shown that SHPT is common among our CKD patients. This study also found no association between SHPT and its clinical features, thus stressing the need for the provision of facilities for the detection of SHPT, which often is not the case in resource limited settings like ours. Our study was limited by the inability to detect skeletal and extra skeletal manifestations of SHPT as well as novel biomarkers like FGF-23.

## Conclusion

SHPT is common in CKD patients in Jos, Nigeria and is independently associated with hypocalcaemia, hyperphosphataemia and elevated alkaline phosphatase levels. It increases with worsening renal function and occurs as early as CKD stage G3a. SHPT is not associated with its clinical features, thus it is important that facilities for the determination of iPTH be made available for the early detection and management of SHPT.
